# The first detection of a serotype O foot‐and‐mouth disease virus in Namibia

**DOI:** 10.1111/tbed.14561

**Published:** 2022-04-25

**Authors:** Frank Banda, Albertina Shilongo, Emmanuel H. Hikufe, Siegfried Khaiseb, Juliet Kabajani, Beatrice Shikongo, Paul Set, Joseph K. Kapapero, Kenneth K. Shoombe, Georgina Zaire, Swithine Kabilika, Melvyn Quan, Elliot M. Fana, Mokganedi Mokopasetso, Joseph M. K. Hyera, Jemma Wadsworth, Nick J. Knowles, Antonello Di Nardo, Donald P. King

**Affiliations:** ^1^ Central Veterinary Research Institute Lusaka Zambia; ^2^ Department of Veterinary Tropical Diseases Faculty of Veterinary Science University of Pretoria, Pretoria South Africa; ^3^ Directorate of Veterinary Services Ministry of Agriculture Water and Land Reform Government Office Park Luther Street Windhoek Namibia; ^4^ Central Veterinary Laboratory Windhoek Namibia; ^5^ Department of Veterinary Services Ministry of Fisheries and Livestock Lusaka Zambia; ^6^ OIE Sub‐Saharan Africa Regional Reference Laboratory (SSARRL) for FMD Botswana Vaccine Institute, Lejara Gaborone Botswana; ^7^ FAO World Reference Laboratory for FMD The Pirbright Institute Pirbright UK

**Keywords:** foot‐and‐mouth disease, serotype O, Southern Africa, transboundary

## Abstract

This report describes the molecular characterization of a serotype O foot‐and‐mouth disease virus (FMDV) recovered from a field outbreak in the Zambezi region, Namibia during July 2021. Sequence analysis demonstrates that this FMDV belongs to the O/EA‐2 topotype sharing closest nucleotide identity (99.5%) to FMD viruses collected since 2018 in Zambia. This is the first detection of serotype O in Namibia, and together with the cases that have been recently detected in southern Zambia, represent the first time that this serotype has been detected in the Southern African FMD endemic pool since 2000, when a virus of Asian origin (O/ME‐SA/PanAsia) caused an outbreak in South Africa. This incursion poses a new threat for the region and the potential onward spread of O/EA‐2 will now need to be closely monitored since serotype O vaccines are not widely used in Namibia, nor in neighbouring countries.

## 1 INTRODUCTION

Foot‐and‐mouth disease (FMD) is caused by a highly contagious virus (FMDV: family *Picornaviridae*, genus: *Aphthovirus*) that infects cloven‐hooved animals. The disease circulates in Asia, Africa and parts of South America where outbreaks lead to livestock production losses and disruption to international trade (Knight‐Jones & Rushton, 2013). In sub‐Saharan Africa, FMD is caused by five different serotypes of FMDV: O, A, Southern African Territories (SAT) 1, SAT 2 and SAT 3 (Casey et al., 2014). Southern Africa comprises FMD endemic pool 6 (Paton et al., 2009), which encompasses Namibia, Botswana, Zimbabwe, Malawi, Mozambique and South Africa where FMD outbreaks reported during the last 20 years have been restricted to those caused by the three SAT serotype viruses (Maree et al., 2014). Zambia, although belonging to this endemic pool, represents a special case as it forms an interface between the Eastern African and Southern Africa endemic pools (Pool 4 and Pool 6, respectively). Prior to 2018, outbreaks due to serotype O were restricted to the Northern Province of Zambia (most recently detected in 2010) where there was close genetic relationship with viral sequences found in other countries in Pool 4. However, since 2018 this serotype has been detected in a number of central and southern Zambian provinces representing a new incursion of serotype O into the Southern African Pool (Banda et al., 2021).

Namibia maintains a ‘FMD‐free without vaccination’ zone officially recognized by the World Organisation for Animal Health (OIE), located to the south of the Veterinary Cordon Fence (VCF). The VCF initially established in 1897 to protect farms in the south of the country from rinderpest, now provides a barrier to protect the high‐value commercial export markets in southern Namibia from FMD that circulates in the eastern Zambezi region (communal grazing land) in the northern part of the country. The Northern Communal Areas of Namibia have no official OIE status for FMD. FMD outbreaks detected recently in the north of Namibia include continued cases due to serotype SAT 2 (topotype III) in 2021 as well as outbreaks due to serotype SAT 3 (topotype II) in 2019 (https://www.wrlfmd.org/southern‐africa/namibia#panel‐4082).

Here, we describe the detection of serotype O in cattle in Namibia which represents the first occurrence of this serotype in the country. New genetically related FMDV sequences for samples collected in Zambia since 2018 are also included in this analysis to help understand the epidemiological connectivity in the region. These cases represent new threats with a potential for onward spread of this lineage to neighbouring countries where serotype O is not present.

## 2 MATERIALS AND METHODS

This report describes new FMD viral sequence data for samples collected from Namibia (2021) and Zambia (2018–21).

FMD cases in Namibia were detected in Zambezi Region of Namibia during June–July 2021. The initial report (on 1 June 2021) involved suspect FMD cases in cattle herd of 144 animals (in Katima Rural Constituency (geo‐coordinates: −17.7816, 24.3347) where 100 (69.4%) of the animals showed clinical signs of FMD including lameness, salivation and frothing of the mouth, blisters in the mouth and hooves. Further suspicion of FMD was subsequently raised in cattle (on 13 July 2021) at Nfooma located in Katima Mulilo Urban (geo‐coordinates: −17.5000, 24.2666) where a total of 70 out of 87 (80.5%) animals exhibited clinical signs indicative of FMD. Vesicular epithelium samples (*n* = 7) collected on 15 July 2021 from Nfooma were submitted to the OIE Sub‐Saharan Africa Regional Reference Laboratory (SSARRL) for FMD (Botswana Vaccine Institute, Gaborone, Botswana) where FMDV diagnosis was carried out as previously described (Teye et al., 2019). The samples were thawed at room temperature, and then blotted dry on absorbent paper. Briefly, a 10 % suspension was prepared by grinding 1 g of the sample in a sterile mortar and pestle with a small volume of tissue culture medium (10% Minimum Essential Medium (MEM 10×), 10% lactalbumin hydrolysate, 4.5% sodium bicarbonate, 1% calf serum, 0.2% penicillin and sterile distilled water). The suspension was clarified by centrifugation at 2000 *g* for 10 min at 4°C, and 500 μl of the suspension was inoculated onto primary lamb kidney cell cultures in 25 cm^2^ culture flasks and incubated for 1 h at 37°C. Fresh cell culture medium (15 ml) was then added and incubated at 37°C and monitored for cytopathic effect (CPE) for 48 h. Samples were considered negative for virus isolation if no CPE was observed after the third passage. An in‐house sandwich ELISA using serotype‐specific rabbit and guinea pig antisera for FMDV serotypes O, A, SAT 1, SAT 2 and SAT 3 (Pirbright Institute, UK; Ferris & Dawson, 1988) was used to detect FMDV antigens and to define the serotype of the CPE positive samples. FMDV sequences encoding VP1 were generated for serotype O positive samples using a published RT‐PCR amplification and sequencing method (Knowles et al., 2016). Briefly, the RT‐PCR amplification used the following primers: O‐1C244F 5ʹ‐GCA GCA AAA CAC ATG TCA AAC ACC TT‐3′, and EUR−2B52R_5ʹ‐GAC ATG TCC TCC TGC ATC TGG TTG AT‐3ʹ and cycle‐sequencing adopted O‐1C244F and NK72 5ʹ‐GAA GGG CCC AGG GTT GGA CTC‐3ʹ.

The 1D nucleotide sequences encoding VP1 (633 nucleotides) were compared with other related serotype O viruses collected from the region. In addition to published sequences, the comparative data included unpublished sequences recently obtained at the FAO World Reference Laboratory for FMD (WRLFMD, Pirbright, UK) for 33 samples received from Zambia. Collection locations and dates for these samples are outlined in Table 1. At the WRLFMD, field isolates were generated in bovine thyroid primary cell cultures and typed using Ag‐ELISA (Ferris & Dawson, 1988). VP1 encoding regions were amplified using two separate RT‐PCR reactions with primers O‐1C244F and EUR‐2B52R as previously described (Banda et al., 2021; Knowles et al., 2016), or with FMD‐3161F and FMD‐4303R (Dill et al., 2017). Cycle sequencing adopted the same primers in separate reactions (Knowles et al., 2016). New sequence data have been submitted to the DDBJ/EMBL/GenBank databases under accession numbers OM259982–OM260015 (Table 1).

**TABLE 1:** Sample details of O/EA‐2 viruses collected recently from Zambia and Namibia (2018–2021)

Virus name	GenBank number	Date collected	Sampling location	Province/region	Country
O/ZAM/1/2018	MZ486060	24/03/2018	Chisamba	Central	Zambia
O/ZAM/2/2018	MZ486061	24/03/2018	Chisamba	Central	Zambia
O/ZAM/3/2018	MZ486062	02/04/2018	Chisamba	Central	Zambia
O/ZAM/1/2019	MZ486065	18/01/2019	Chisamba	Central	Zambia
O/ZAM/2/2019	MZ486066	11/02/2019	Ufwenuka, Monze	Southern	Zambia
O/ZAM/3/2019	MZ486067	11/02/2019	Ufwenuka, Monze	Southern	Zambia
O/ZAM/4/2019	MZ486068	11/03/2019	Magoye, Mazabuka	Southern	Zambia
O/ZAM/7/2019	MZ486069	30/03/2019	Kasako, Mazabuka	Southern	Zambia
O/ZAM/8/2019	MZ486070	30/03/2019	Kasako, Mazabuka	Southern	Zambia
O/ZAM/15/2019	OM259982^b^	31/05/2019	Namwala	Southern	Zambia
O/ZAM/16/2019	OM259983^b^	31/05/2019	Namwala	Southern	Zambia
O/ZAM/18/2019	OM259984^b^	05/06/2019	Monze	Southern	Zambia
O/ZAM07/2019^a^	MZ486072	28/06/2019	Musenga, Chingola	Copperbelt	Zambia
O/ZAM/20/2019	OM259985^b^	28/06/2019	Makeni	Lusaka	Zambia
O/ZAM/21/2019	OM259986^b^	28/06/2019	Makeni	Lusaka	Zambia
O/ZAM/24/2019	OM259987^b^	17/07/2019	Kafue	Lusaka	Zambia
O/ZAM/26/2019	OM259988^b^	17/07/2019	Chibombo	Central	Zambia
O/ZAM/27/2019	OM259989^b^	17/07/2019	Chibombo	Central	Zambia
O/ZAM/28/2019	OM259990^b^	07/08/2019	Chikankata	Southern	Zambia
O/ZAM/54/2019	OM259991^b^	01/09/2019	Chikankata	Southern	Zambia
O/ZAM/55/2019	OM259992^b^	06/09/2019	Kabwe	Central	Zambia
O/ZAM/56/2019	OM259993^b^	06/09/2019	Kabwe	Central	Zambia
O/ZAM/57/2019	OM259994^b^	01/10/2019	Mpande	Northern	Zambia
O/ZAM/58/2019	OM259995^b^	01/10/2019	Sinazongwe	Southern	Zambia
O/ZAM/60/2019	OM259996^b^	17/10/2019	Kasempa	North‐western	Zambia
O/ZAM/62/2019	OM259997^b^	01/12/2019	Itezi tezi	Central	Zambia
O/ZAM/1/2020	OM259998^b^	05/02/2020	Chilanga	Lusaka	Zambia
O/ZAM/2/2020	OM259999^b^	05/02/2020	Chilanga	Lusaka	Zambia
O/ZAM/3/2020	OM260000^b^	16/03/2020	Namwala	Southern	Zambia
O/ZAM/4/2020	OM260001^b^	16/03/2020	Namwala	Southern	Zambia
O/ZAM/6/2020	OM260002^b^	15/05/2020	Kalomo	Southern	Zambia
O/ZAM/7/2020	OM260003^b^	15/05/2020	Kalomo	Southern	Zambia
O/ZAM/8/2020	OM260004^b^	10/08/2020	Kazungula	Southern	Zambia
O/ZAM/9/2020	OM260005^b^	10/08/2020	Kazungula	Southern	Zambia
O/ZAM/10/2020	OM260006^b^	10/08/2020	Kazungula	Southern	Zambia
O/ZAM/11/2020	OM260007^b^	18/08/2020	Makeni	Lusaka	Zambia
O/ZAM/12/2020	OM260008^b^	18/08/2020	Makeni	Lusaka	Zambia
O/ZAM/14/2020	OM260009^b^	28/10/2020	Nkeyema	Western	Zambia
O/ZAM/15/2020	OM260010^b^	28/10/2020	Nkeyema	Western	Zambia
O/ZAM/16/2020	OM260011^b^	28/10/2020	Nkeyema	Western	Zambia
O/ZAM/7/2021	OM260012^b^	18/03/2021	Kalomo	Southern	Zambia
O/ZAM/9/2021	OM260013^b^	08/05/2021	Mwandi	Western	Zambia
O/ZAM/11/2021	OM260014^b^	12/05/2021	Mwandi	Western	Zambia
O/NAM10/2021^a^		15/07/2021	Nfooma, Katima Mulilo	Zambezi	Namibia
O/NAM11/2021^a^		15/07/2021	Nfooma, Katima Mulilo	Zambezi	Namibia
O/NAM13/2021^a^	OM260015^b^	15/07/2021	Nfooma, Katima Mulilo	Zambezi	Namibia
O/NAM14/2021^a^		15/07/2021	Nfooma, Katima Mulilo	Zambezi	Namibia

NB: All samples were collected from cattle except ZAM/1/2020, ZAM/2/2020 and ZAM/18/2019 which were from pigs.

^a^ Not a WRLFMD isolate number.

^b^ New sequence data presented in this report.

The complete VP1 encoding nucleotide sequences were aligned using BioEdit 7.0.5.3 (Hall, 1999) and Clustal W 1.83 (Thompson et al., 1994). To construct a maximum likelihood (ML) phylogeny (Nei & Kumar, 2000), the data set was tested for 24 common nucleotide substitution models using MEGA 7 (Kumar et al., 2016). The model with the lowest Bayesian information criterion (BIC) score (Tamura 3‐parameter with gamma distribution; Tamura, 1992) was chosen to construct the tree. The robustness of the tree topology was assessed with 1000 bootstrap pseudo‐replicates.

## 3 RESULTS AND DISCUSSION

**FIGURE 1:**
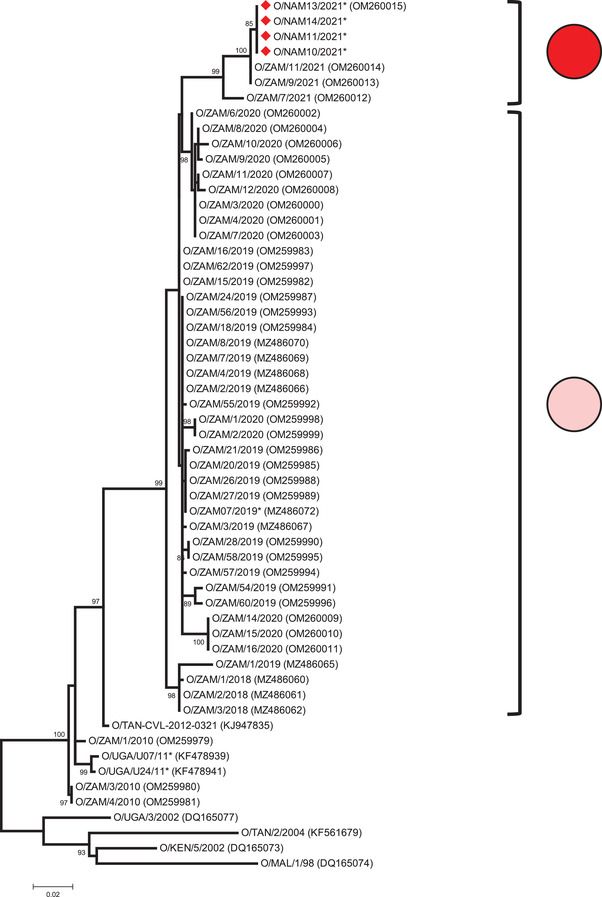
A midpoint‐rooted maximum likelihood tree highlighting the monophyletic relationship of O/EA‐2 FMD viruses collected from Zambia and Namibia during 2018–2021. Red diamonds highlight FMDV sequences from Namibia and coloured dots represent data for samples that were sequenced during 2021 (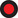) and 2018–2020 (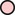). Bootstrap values of 70% and above are shown

Testing at BVI, Botswana, yielded FMDV positive results for five out of seven of the Namibian samples using virus isolation which were all characterized as serotype O using antigen ELISA. The sequences encoding VP1 of four selected virus isolates from Namibia (O/NAM10/2021, O/NAM11/2021, O/NAM13/2021 and O/NAM14/2021) were identical to each other and were categorized as belonging to the EA‐2 topotype within serotype O. This analysis showed that the Namibian sequences shared closest nucleotide identity (99.5%) to two FMDV isolates that had been collected from the Western Province of Zambia during 2021 (O/ZAM/9/2021 and O/ZAM/11/2021; Figure 1).

The O/EA‐2 topotype originates from East Africa (FMD endemic Pool 4; Paton et al., 2009) where it has been detected in countries such as Burundi, Democratic Republic of the Congo, Kenya, Tanzania and Uganda (Kasanga et al., 2015; Kerfua et al., 2019; Namatovu et al., 2015; Wekesa et al., 2015). The O/EA‐2 topotype has also previously appeared transiently into the northern parts of some Southern African countries such as Malawi where an isolate collected in 1998 shared a close genetic relationship to FMD viruses from Tanzania (Kasanga et al., 2015). The first confirmed serotype O outbreak in Zambia was in Mbala District of Northern Province in 1976 (Perry & Hedger, 1984). Prior to 2018, serotype O outbreaks in Zambia were restricted to the Northern Province where they were recorded on three occasions (1982, 2000 and 2010). Sequence data for VP1 encoding sequences for O/EA‐2 viruses collected in 2010 close to the border with Tanzania are shown in Figure 1 (O/ZAM1/2010, O/ZAM/3/2010 and O/ZAM/4/2010; Banda et al., 2021).

**FIGURE 2:**
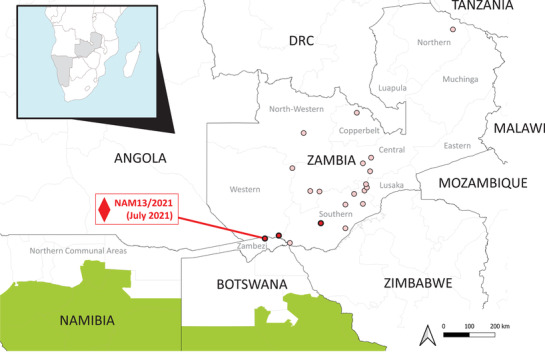
FMD outbreaks caused by the O/EA‐2 topotype that have occurred in Southern African countries since 2018. Locations represent outbreaks from which FMD viruses were sequenced during 2021 (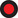) and 2018–2020 (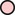), where (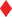) denotes the location of the FMD report in Zambezi Region, Namibia. NB: All FMD outbreaks due to the O/EA‐2 topotype that have been detected in Zambia since 2018 are plotted on the map, although multiple samples and sequences have been collected at some of the locations. Namibia and Botswana maintain FMD‐free zones (without vaccination) which are highlighted in green

Since 2018, O/EA‐2 viruses within a new genetic clade have spread across Zambia to cause outbreaks in Central, Copperbelt, Lusaka, North‐Western, Southern and Western Provinces (Figure 2; Banda et al., 2021). The precise transmission pathways for the introduction of O/EA‐2 into central, southern and western parts of Zambia are not completely understood, but it has been previously suggested that the closure of an abattoir in Mbala District of the Northern Province may have incentivized stock owners to move animal southwards in search of market access (Banda et al., 2021).

Phylogenetic analyses (Figure 1) highlight the transboundary connectivity between Zambia and Namibia and understanding epidemiological connections that underpin the recent spread of the O/EA‐2 topotype in Zambia and Namibia is a priority for new studies. By themselves, these sequence analyses cannot reconstruct the specific epidemiological routes by which O/EA‐2 has been introduced into Namibia; however, the Katima Mulilo Bridge which was built in 2004 to carry the Trans‐Caprivi‐Highway (Walvis Bay‐Ndola‐Lubumbashi Development Road) across the Zambezi River could provide an obvious pathway. Across the border in Zambia, the proximity to international border crossing points and major roads were identified in a previous study as risk factors for FMD outbreaks (Hamoonga et al., 2014). Further work is now required to quantify the risks associated with these transboundary animal movements and to develop controls that reduce the likelihood of further FMD incursions into the region.

The detection of O/EA‐2 in the Zambezi Region located in the Caprivi Strip in north‐east Namibia represents the first time that serotype O has been detected in the country. This incursion now poses a new threat to the FMD endemic region of Namibia and other countries in the region, such as Angola, Botswana and Zimbabwe. Botswana also maintains an OIE official FMD‐free without vaccination zone in the south, but like Namibia has no OIE status for the northern part of the country. The introduction of a new serotype or an antigenically novel variant can pose challenges for regional FMD control initiatives as was recently documented by the rapid spread of the A/ASIA/G‐VII lineage in Asia (Bachanek‐Bankowska et al., 2018). In FMD endemic regions of Southern Africa, onward transmission of O/EA‐2 may go unnoticed due to the significant under‐reporting of the disease. In these areas, serotype O‐specific immunity is likely to be very low since FMD vaccines, where these are used in Southern Africa countries, do not typically contain a serotype O component.

In summary, these findings highlight a new FMD threat for countries in Southern Africa and motivate field teams and laboratory scientists to increase surveillance within the region to rapidly detect further outbreaks due to this topotype. Now that this serotype has been identified, new resources will be required to ensure that laboratories in the region can correctly identify new clinical cases due to serotype O as well as detect serotype O‐specific antibodies in sera collected as part of surveillance activities. Furthermore, FMD vaccines selected for use in the region should now consider these risks, and vaccination campaigns may need to absorb the extra costs of adding a serotype O antigen component to the vaccines that are deployed.

## CONFLICT OF INTERESTS

The authors declare no conflict of interest.

## Data Availability

The data that support the findings of this study are openly available in GenBank at https://www.ncbi.nlm.nih.gov/nuccore, reference numbers OM259982–OM260015.
